# Clinical Evaluation of a Tandem qPCR-CRISPR Assay for Multidrug-Resistant or Rifampicin-Resistant Tuberculosis: A Prospective Diagnostic Accuracy Study

**DOI:** 10.3390/microorganisms14081750

**Published:** 2026-08-09

**Authors:** Ying Wang, Zhe Song, Jianfeng Zeng, Yajuan Dong, Houming Liu, Jian Zeng, Xuhui Liu, Liang Fu, Xuelin Li, Shuihua Lu, Zhen Huang, Xiao-Yong Fan

**Affiliations:** 1 Shanghai Institute of Infectious Disease and Biosecurity, Fudan University, Shanghai 200032, China; yingwang23@m.fudan.edu.cn (Y.W.);; 2 Shanghai Public Health Clinical Center, Fudan University, Shanghai 200032, China; 3 National Clinical Research Center for Infectious Disease, Shenzhen 518112, China; 13823194373@163.com (Z.S.); zhenhuang@tulane.edu (Z.H.); 4 Pulmonary Diseases Department, Shenzhen Third People’s Hospital, Shenzhen 518112, China; 5 Department of Clinical Laboratory, Shenzhen Third People’s Hospital, Shenzhen 518112, China

**Keywords:** tuberculosis, drug resistance, CRISPR, diagnostics

## Abstract

CRISPR-based assays have shown the potential to enhance the diagnosis of tuberculosis (TB), but evidence remains limited owing to scarce independent validation. We prospectively evaluated a tandem qPCR-CRISPR assay detecting *Mycobacterium tuberculosis* (*Mtb*) and rifampicin resistance. Respiratory specimens for smear microscopy, mycobacterial culture, Xpert MTB/RIF Ultra (Xpert) and the investigational assay were collected from suspected TB patients. Phenotypic drug susceptibility testing (pDST) was carried out on culture-positive cases. Diagnostic performance was evaluated against the microbiological reference standard (MRS) and composite reference standard (CRS). The predictive performance for drug resistance was assessed using pDST and Xpert as reference standards. A total of 179 adults were enrolled, including 84 confirmed TB, 18 clinical TB, and 77 non-TB cases. The tandem qPCR-CRISPR assay showed 84.5% (95% CI 75.0–91.5%) sensitivity and 95.8% (95% CI 89.6–98.8%) specificity against MRS, and 70.6% (95% CI 60.6–79.0%) sensitivity and 96.1% (95% CI 88.3–99.0%) specificity against CRS. For drug resistance, the assay achieved a sensitivity of 77.5% (95% CI 61.1–88.6%) and a specificity of 90.9% (95% CI 77.4–97.0%). Sensitivity was higher in sputum than in bronchoalveolar lavage fluid. The tandem qPCR-CRISPR assay showed suboptimal performance for detecting TB and MDR/RR-TB and requires further optimization before clinical application.

## 1. Introduction

Tuberculosis (TB) is the leading cause of death among infectious diseases globally, with approximately 10 million incident cases and 1.5 million deaths annually [1]. Timely and accurate diagnosis is essential for initiating appropriate treatment, preventing transmission, and reducing mortality, particularly for multidrug-resistant or rifampicin-resistant TB (MDR/RR-TB) [1,2]. Despite recent advances, substantial diagnostic challenges remain, especially in high-burden settings [3,4].

Currently, TB diagnosis relies predominantly on conventional methods. Although rapid and inexpensive, acid-fast bacillus (AFB) smear microscopy has limited sensitivity, particularly in paucibacillary specimens [5]. Regarded as the gold standard, the turnaround time of mycobacterial culture is slow, thereby delaying therapy. GeneXpert MTB/RIF Ultra (Xpert), a fully automated PCR-based platform targeting the *rpoB* gene, enables simultaneous detection of *Mycobacterium tuberculosis* (*Mtb*) and rifampicin (RIF) resistance within a two-hour timeframe [6]. However, Xpert requires specialized equipment with an integrated thermocycler and proprietary single-use cartridges [7].

Clustered regularly interspaced short palindromic repeats (CRISPR) and CRISPR-associated (Cas) proteins form an adaptive immune system in bacteria and archaea: fragments of invading phage or plasmid DNA are archived between palindromic repeats and transcribed into CRISPR RNAs (crRNAs) that guide Cas nucleases to cleave matching foreign nucleic acids [8]. Diagnostics repurpose this guide-directed recognition. Once a crRNA binds its target, Cas12a is activated and indiscriminately cleaves single-stranded DNA reporters in the reaction, converting a single recognition event into a fluorescence signal [9]. CRISPR-based diagnostics (CRISPR-Dx) represent a promising approach to address current gaps in accessibility and performance, leveraging their high sensitivity and specificity when combined with nucleic acid amplification strategies [10,11,12]. These platforms can detect *Mtb* DNA across diverse specimen types, including blood [13], sputum [14,15], bronchoalveolar lavage fluid (BALF) [15], and pleural effusions [16]. Beyond specimen versatility, CRISPR-Dx operates isothermally or near-isothermally with minimal instrumentation, returns results within 1–2 h, and costs little per test. Its crRNAs can also be reprogrammed to add targets or resistance mutations without re-designing the hardware. Cartridge-based platforms such as Xpert cannot match this flexibility, because they require a dedicated thermocycler and proprietary single-use cartridges. Compared with culture, CRISPR-Dx offers substantially reduced turnaround time. These advantages, however, remain largely unproven in clinical cohorts, which is the gap the present study addresses. Most CRISPR-based TB diagnostics remain at the proof-of-concept stage or are limited by small sample sizes, with a lack of prospective clinical evaluation.

In this study, we conducted a prospective evaluation of a tandem qPCR-CRISPR assay that integrates qPCR-based TB diagnosis with CRISPR-mediated identification of MDR/RR-TB, and independently evaluated its performance. Our findings provide preliminary evidence for its potential clinical utility, revealing that the technique remains at an early developmental stage and requires further optimization and large-scale validation before clinical implementation.

## 2. Materials and Methods

### 2.1. Study Design and Participants

Adults with suspected TB who visited the pulmonary clinic at Shenzhen Third People’s Hospital between 1 January 2024 and 31 August 2024 were consecutively enrolled. Sputum and/or BALF specimens were collected for routine clinical testing and the tandem qPCR-CRISPR assay. All participants underwent Xpert MTB/RIF Ultra testing (Cepheid, Sunnyvale, CA, USA); most additionally received AFB smear microscopy and *Mtb* culture. Imaging examinations were performed when necessary, and medical records were reviewed to document symptoms and verify the microbiological results.

Participants with incomplete clinical data, insufficient specimen volume, or prior initiation of anti-TB therapy before specimen collection were excluded. Bacteriologically confirmed TB cases were patients from whom a biological specimen had tested positive by smear microscopy, culture or Xpert. Cases with resistance to rifampicin detected by phenotypic drug susceptibility testing (pDST) or Xpert were classified as MDR/RR-TB. pDST and Xpert were combined because of failure rates and prolonged turnaround times. Consequently, not all specimens in this study yielded valid pDST results. Xpert MTB/RIF Ultra, a WHO-endorsed molecular test, served as the alternative reference. Restricting the resistance analysis to pDST would have excluded culture-negative but Xpert-confirmed MDR/RR-TB cases and reduced the analysable sample below the calculated requirement. Clinically diagnosed TB cases referred to patients who did not meet the criteria for bacteriological confirmation but were diagnosed with active TB by a clinician, who subsequently initiated a full course of anti-TB treatment [4]. Participants ultimately determined to be free of TB were selected as non-TB controls.

The study protocol was approved by the Human Ethics Review Committee of Shenzhen Third People’s Hospital (2023-051 and 2024-005), and written informed consent was obtained from all participants.

### 2.2. Specimen Collection and Processing

Fresh sputum (≥0.5 mL) was collected from all participants. For patients who underwent bronchoscopy and had BALF collected, remaining BALF samples were also obtained. All specimens were stored at −80 °C and processed within one month. Each specimen was liquefied with twice the volume of 4% NaOH and incubated at room temperature for 30 min. A 1 mL aliquot of the liquefied specimen was centrifuged at 12,000 rpm for 5 min. Precipitates were resuspended in 100 μL of supernatant. The 10 µL of internal control derived from *Bacillus subtilis* was added to the sediment to monitor the extraction and amplification procedures.

DNA was extracted from the specimens using a manufacturer-matched magnetic bead-based kit (TOLOBIO, Wuxi, China). Then 800 µL of lysis buffer (containing guanidine thiocyanate, sodium chloride, and EDTA) and 30 µL of magnetic beads were added to the resuspension solution and vortexed for 2 min. Beads were captured by magnetic separation, sequentially washed with 700 μL Wash Buffer 1 (mainly sodium chloride, EDTA, and Tris-HCl) and Wash Buffer 2 (mainly purified water), and then air-dried at room temperature for 2 min. Next, the beads were resuspended in 50 μL of elution buffer and vortexed for 1 min to release the bound nucleic acids. The supernatants were then collected for downstream applications.

### 2.3. GeneXpert MTB/RIF Ultra Testing

Xpert testing was performed according to the manufacturer’s instructions. Prior to processing, sputum specimens were inspected to ensure the absence of food particles or other solid debris. Sputum specimens were mixed with twice their volume of sample reagent (SR), whereas BALF specimens were mixed with an equal volume of SR. The mixtures were vigorously shaken 10–20 times or vortexed for at least 10 s and incubated at room temperature for 10 min. The samples were then subjected to an additional round of thorough shaking or vortexing, followed by a further 5-min incubation to ensure complete liquefaction. Processed specimens were tested as promptly as possible. Using a disposable pipette, 2 mL of the liquefied sample was transferred to the sample chamber of a cartridge, and the GeneXpert-Dx instrument (Cepheid, Sunnyvale, CA, USA) was initiated to run the assay. Upon completion, results were retrieved directly from the instrument’s report interface. Each run incorporated an internal sample processing control (SPC) and a probe check control (PCC) to verify adequate sample processing and reagent integrity. Results for *Mtb* were reported as high, medium, low, very low, trace, or not detected. For *Mtb*-positive specimens, rifampicin resistance was reported as “Rifampicin resistance detected,” “Not detected,” or “Indeterminate”.

### 2.4. Phenotypic Drug Susceptibility Testing

pDST was performed using the Sensititre^®^ *Mycobacterium tuberculosis* MIC plate (Thermo Fisher Scientific, Shanghai, China) according to the manufacturer’s instructions. The plate is a 96-well microtiter format containing serial dilutions of 12 antibiotics and two positive growth control wells, including rifampicin at concentrations ranging from 0.12 to 16 μg/mL. Single colonies of *Mtb* isolated on medium were emulsified in 0.2% Tween-80 saline containing glass beads and vortexed for at least 30 s to ensure uniform suspension. Following a 15-min settling period, the turbidity was adjusted to a 0.5 McFarland standard. One hundred microliters of the bacterial suspension was transferred to Middlebrook 7H9 broth containing Oleic Albumin Dextrose Catalase Supplement (OADC) and vortexed for 30 s to achieve a final inoculum of 1 × 10^5^ CFU/mL (acceptable range: 5 × 10^4^–5 × 10^5^ CFU/mL). One hundred microliters of the prepared inoculum was dispensed into each well of the MIC plate. The entire inoculation procedure was completed within 30 min. The plate was sealed with adhesive film, surface-disinfected, and incubated aerobically at 35–37 °C for 10 days. If growth was insufficient at day 10, incubation was extended for an additional 11 days. Results were read using the Sensititre^®^ Manual Viewer system (Thermo Fisher Scientific, Shanghai, China). The minimum inhibitory concentration (MIC) was defined as the lowest antibiotic concentration at which growth was distinctly inhibited. A strain was classified as resistant to a given drug if its MIC exceeded the critical concentration, and as susceptible if the MIC was less than or equal to the critical concentration. The critical concentration for rifampicin resistance was set at 0.5 μg/mL, recommended by WHO since the 2021 revision.

### 2.5. The Tandem qPCR-CRISPR Assay

The tandem qPCR-CRISPR assay was supplied as a research-use-only kit that was not commercially available, and manufacturer information was not disclosed. All components, including primers, probes, enzymes, and buffers used in the assay, were supplied in the kit. The developer disclosed the analytical validation data indicating a limit of detection of 10 CFU/mL for *Mtb* and 100 CFU/mL for rifampicin resistance mutation detection in spiked sputum and no cross-reactivity with 15 non-*Mtb* species or 15 common respiratory pathogens. It can detect ≥5% of resistant strains in mixed bacterial populations at concentrations of ≥1 × 10^3^ CFU/mL. Intra-assay testing across 10 replicates of reference materials yielded a coefficient of variation (CV) ≤ 15%. All primers and crRNAs used in the assay are available in Supplementary Table S1.

The assay was designed to target *IS6110*, a multi-copy sequence within the genome, for *Mtb* detection by qPCR. Drug resistance was identified by CRISPR-mediated detection targeting mutations at codons 516, 526 and 531 of the *rpoB* gene, which together account for majority of rifampicin-resistant *Mtb* isolates [17,18,19]. This restricted three-crRNA panel reflects the developer’s design priority of a compact, simpler format and greater clinical applicability rather than exhaustive mutation coverage. The investigational assay was performed by personnel blinded to all clinical information; reciprocally, technologists performing Xpert, culture, and pDST were unaware of the qPCR-CRISPR results. The two workflows were conducted in physically separate laboratories on independently aliquoted portions of the same specimen. Formal repeatability and reproducibility experiments were not performed, because the study was designed as a clinical accuracy evaluation of a single-run testing algorithm reflecting routine practice. The assay was performed as follows:

qPCR-based detection of the *Mtb*: The 5 μL of extracted DNA solution was combined with 9.7 μL of PCR master mix and 0.3 μL of polymerase mix to form the final reaction mixture. The qPCR protocol was as follows: 50 °C for 10 min, 98 °C for 30 s, and 45 cycles of 98 °C for 5 s and 61 °C for 20 s. Fluorescence signals were recorded in the VIC and Cy5 channels at 61 °C. An internal control (Cy5) cycle threshold (Ct) < 35 indicated valid extraction and amplification. A sample was considered positive for *Mtb* if the VIC channel Ct value was ≤40.

CRISPR-based detection of drug resistance: Amplicons from *Mtb*-positive specimens were further tested by a CRISPR/Cas12a system. Each 20 μL reaction mixture contained 16.5 μL of reaction buffer with ssDNA probes, 1 μL of crRNA mixture, 0.5 μL of Cas12a enzyme, and 2 μL of qPCR product. Reactions were incubated at 48 °C for 20 cycles (30 s per cycle), with FAM fluorescence signals recorded. The resistance index R(20)/R(1) was calculated, where R(20) and R(1) represented fluorescence intensities at cycle 20 and cycle 1, respectively. R(20)/R(1) ≥ 4.0 indicated drug resistance. The complete workflow, including decision points and interpretation rules, is illustrated in Supplementary Figure S1.

Quality control: Every run included a positive control (inactivated *IS6110* and S531L DNA), a no-template control, and the *B. subtilis* internal control; a run was invalidated if any control failed.

### 2.6. Statistical Analysis

According to previous research reported by WHO [20], the minimum acceptable performance thresholds were set at 80% sensitivity and 95% specificity, while the assay under evaluation was expected to attain 92% and 100%, respectively. At a two-sided α of 0.05 and β = 0.2, the sample size calculation indicated a minimum requirement of 65 positive and 73 negative specimens.

Diagnostic sensitivity, specificity, positive predictive values (PPVs), negative predictive values (NPVs), positive likelihood ratio (PLR), and negative likelihood ratio (NLR) together with their 95% confidence intervals (CIs) were estimated against the microbiological reference standard (MRS) and composite reference standard (CRS). The MRS was a positive result on AFB smear microscopy, mycobacterial culture, or Xpert from at least one respiratory specimen. The CRS comprised the MRS together with clinically diagnosed TB, defined as active TB incorporating clinical presentation together with histological or radiological evidence. Continuous variables were presented as medians accompanied by interquartile ranges (IQRs), and categorical variables as frequencies with percentages. Continuous variables were compared by the Kruskal–Wallis test and categorical variables by chi-square or Fisher’s exact tests. Cohen’s kappa (κ) was used to quantify agreement between the reference standards. All statistical tests were two-sided. *p*-values less than 0.05 were considered statistically significant. Statistical analyses were done using SPSS statistical software version 25. Data visualization was performed using Prism GraphPad version 9.4.1.

## 3. Results

### 3.1. Participant Characteristics

**Figure 1:**
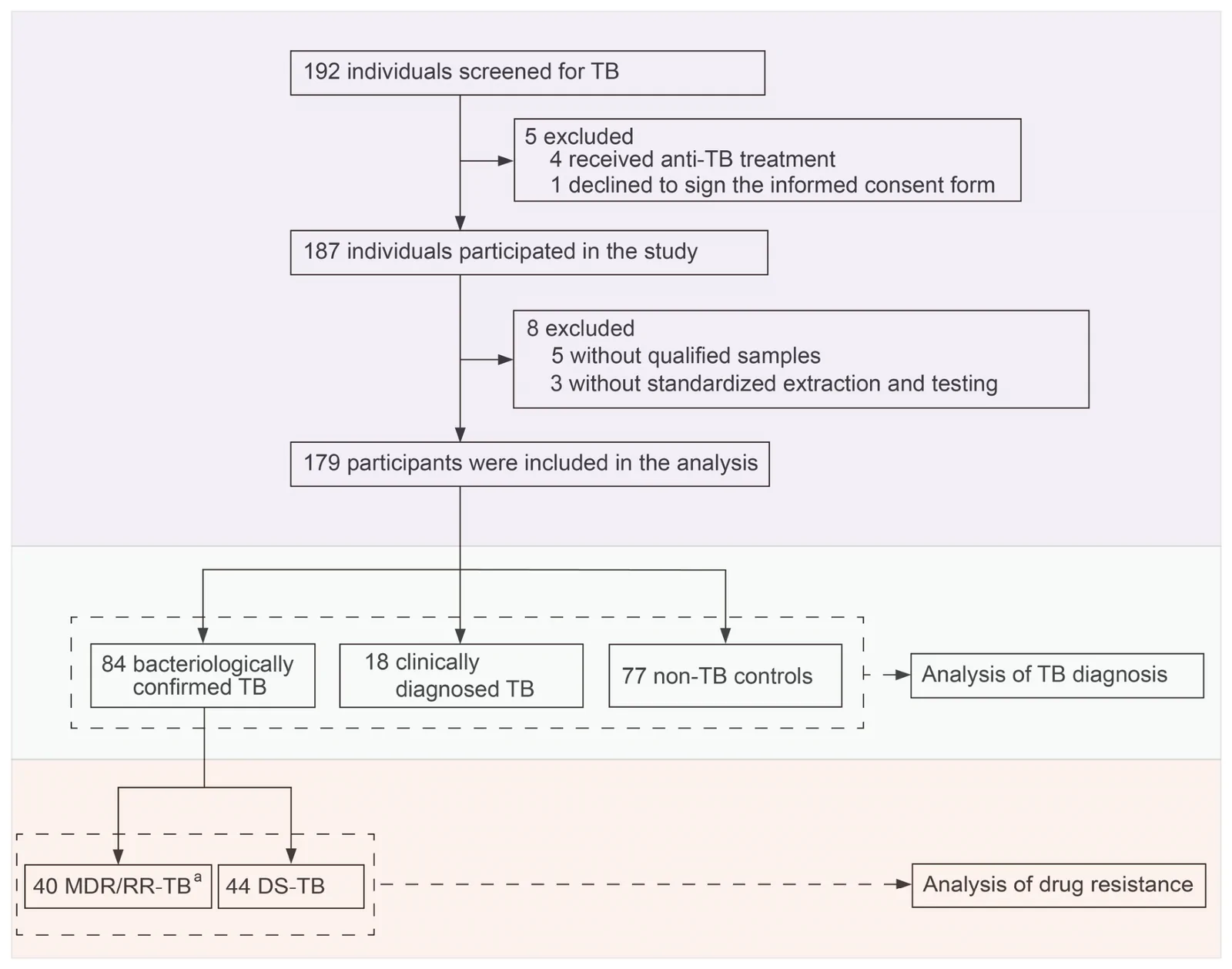
Study participants. ^a^ Any positive result of drug resistance by Xpert or pDST was considered MDR/RR-TB; participants were not required to have had all reference tests undertaken. Definition of abbreviations: TB =Tuberculosis; MDR/RR-TB = Multi-drug-resistant or rifampicin-resistant TB; DS-TB = Drug-susceptible tuberculosis.

During the study period, 192 adults with suspected TB presented to the clinic, of whom 187 consented to participate. Eight participants were excluded from analysis: five due to insufficient specimen volume and three due to nucleic acid contamination. A total of 179 participants were included in the final analysis, comprising 84 bacteriologically confirmed, 18 clinically diagnosed, and 77 non-TB cases. Among confirmed cases, 40 were classified as MDR/RR-TB and 44 as drug-susceptible TB (DS-TB) (Figure 1). All participants had a median age of 52 years (IQR 35.0–60.0), and 57 (31.8%) were female. Although nearly all patients exhibited typical TB manifestations, only 96 (53.6%) were positive by interferon-gamma release assay (IGRA), 48 (26.8%) by smear, 67 (37.4%) by culture, and 78 (43.6%) by Xpert (Table 1).

### 3.2. Diagnostic Performance for TB Detection

The tandem qPCR-CRISPR assay correctly identified 71 (84.5%) bacteriologically confirmed TB cases and 1 (5.6%) clinically diagnosed case, with 3 (3.9%) false-positive results among non-TB cases. Against the MRS, the assay demonstrated a sensitivity of 84.5% (95% CI 75.0–91.5%) and a specificity of 95.8% (95% CI 89.6–98.8%), with a PPV of 94.7% (95% CI 87.1–98.2%) and an NPV of 87.5% (95% CI 85.2–94.4%). The PLR and NLR were 20.1 (95% CI 7.7–52.6) and 0.2 (95% CI 0.1–0.3). Against the CRS, the overall sensitivity was 70.6% (95% CI 60.6–79.0%), and the specificity was 96.1% (88.3–99.0%), with PPV and NPV of 96.0% (95% CI 88.0–99.0%) and 71.2% (95% CI 61.3–79.4%), respectively (Table 2). The corresponding PLR and NLR were 18.1 (95% CI 6.0–55.3) and 0.3 (95% CI 0.2–0.4).

**Figure 2:**
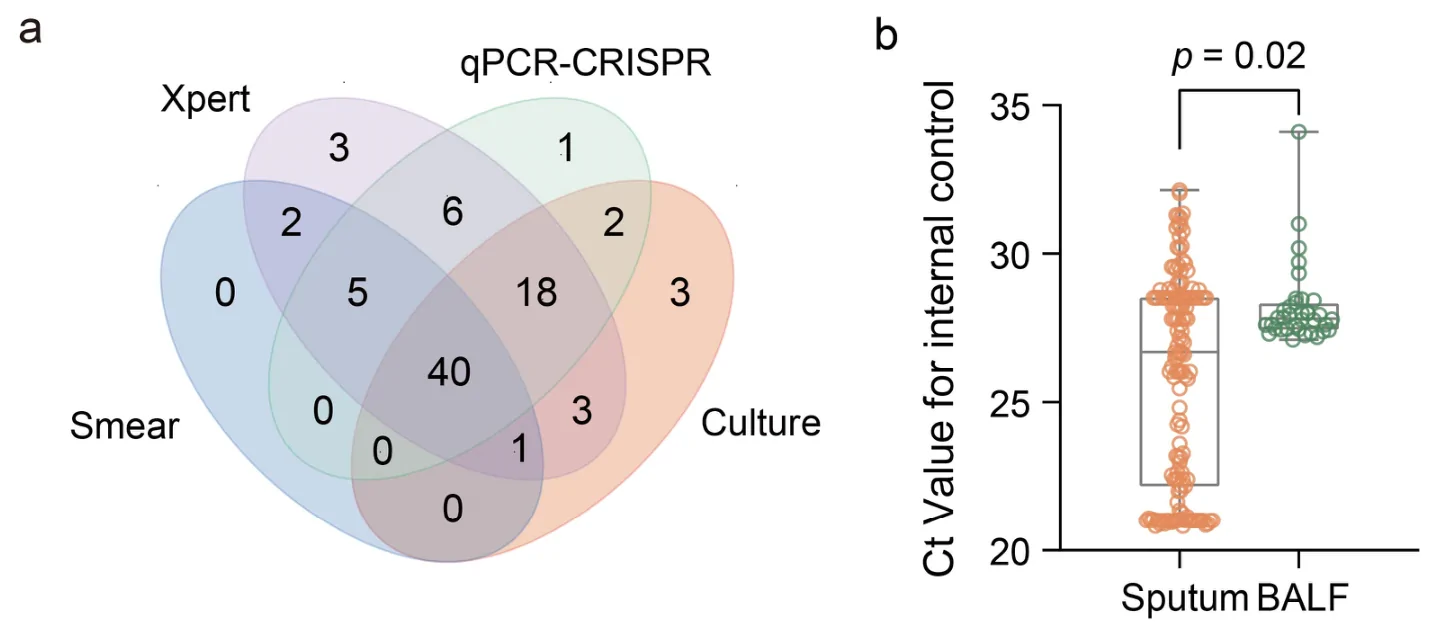
Diagnostic performance of tandem qPCR–CRISPR assay for TB detection. (**a**) A Venn diagram illustrates positive results from smear, mycobacterial culture, Xpert, and tandem qPCR–CRISPR assay in TB cases (including bacteriologically confirmed TB and clinically diagnosed TB). (**b**) The Ct values of the internal control from sputum and BALF specimens. Definition of abbreviations: TB = Tuberculosis; Xpert = GeneXpert MTB/RIF Ultra; Ct = cycle threshold; BALF = bronchoalveolar lavage fluid.

The tandem qPCR-CRISPR assay showed strong concordance with Xpert (κ = 0.829, *p* < 0.001) (Figure 2a) and detected 48.9% (22/45) of smear-negative and 22.2% (6/27) of culture-negative cases (Table 2). However, diagnostic performance varied by specimen type, with higher sensitivity in sputum than in BALF [77.6% (59/76) vs. 50.0% (13/26)], whereas specificity was comparable [95.5% (64/67) vs. 100.0% (10/10)] (Table 2). This pattern was accompanied by significantly lower internal-control Ct values in sputum than in BALF (median 26.70 vs. 27.82) (Figure 2b).

### 3.3. Performance of Drug Resistance Detection

**Figure 3:**
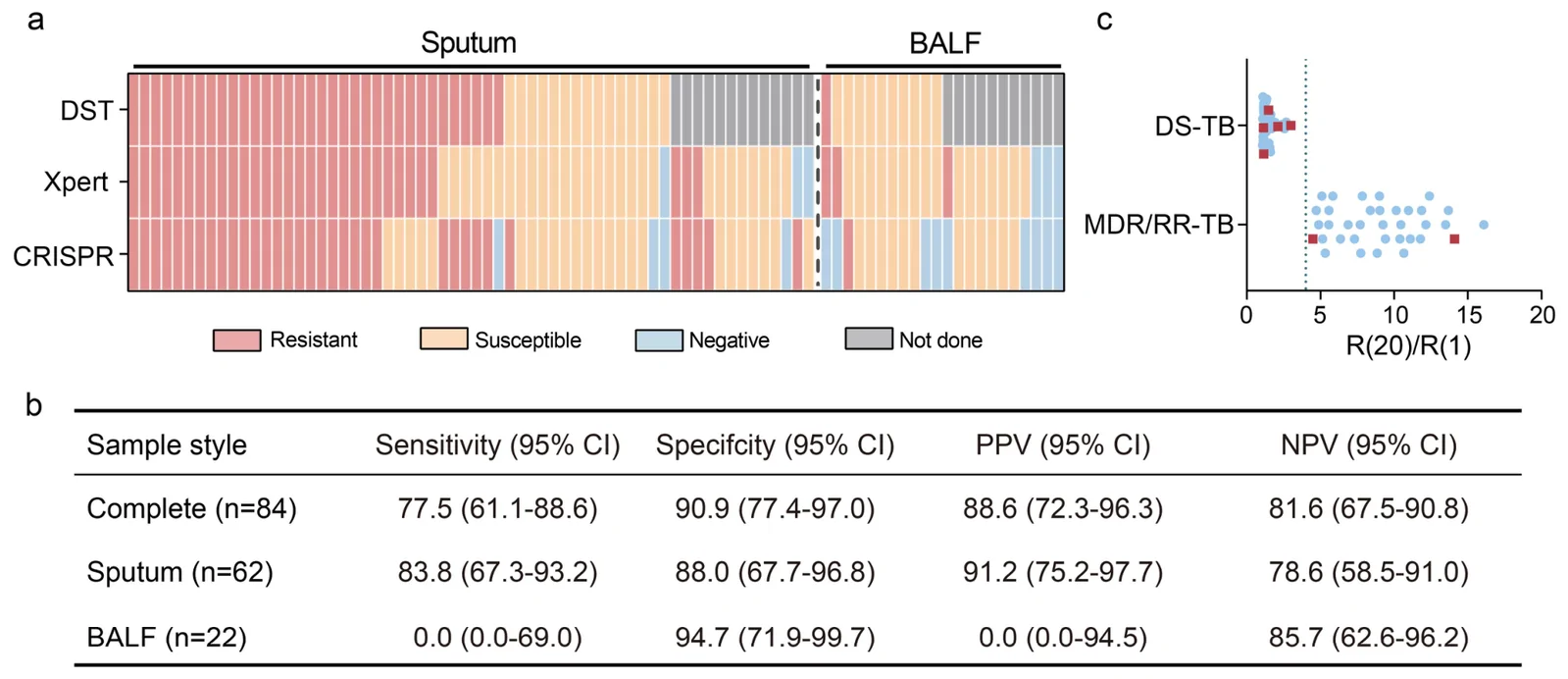
Performance of the tandem qPCR–CRISPR assay in detecting MDR/RR-TB. (**a**) Heat map of drug resistance results for confirmed tuberculosis (TB) cases. The sample types include sputum and BALF. The heat map shows samples represented in columns and assigned testing methodologies in rows. (**b**) The performance of the tandem qPCR–CRISPR assay for detecting MDR/RR-TB. All tests were performed on the same 84 bacteriologically confirmed TB cases. All values are presented as percent (95% CI). (**c**) Scatter plots depict the distribution of R(20)/R(1) values from tandem qPCR–CRISPR assay testing of *Mtb*-positive cases. R(20) and R(1) represent fluorescence intensities at cycle 20 and cycle 1 of the drug resistance detection curve, respectively. The blue dots indicate the true-positive and true-negative results. The red square dots indicate the false-negative and false-positive results. The blue dashed line indicates the threshold for a positive signal. Definition of abbreviations: pDST = Phenotypic drug susceptibility testing; Xpert = GeneXpert MTB/RIF Ultra; BALF = Bronchoalveolar lavage fluid; DS-TB = Drug-susceptible tuberculosis; MDR/RR-TB = Multidrug-resistant or rifampicin-resistant TB; PPV = positive predictive value; NPV = negative predictive value; CI = confidence intervals.

The performance for detecting drug resistance was evaluated in 84 bacteriologically confirmed TB cases against pDST and/or Xpert as the reference (Figure 1). The tandem qPCR-CRISPR assay demonstrated a sensitivity of 77.5% (95% CI 61.1–88.6%) and a specificity of 90.9% (95% CI 77.4–97.0%) for MDR/RR-TB identification, with a PPV of 88.6% (95% CI 72.3–96.3%) and NPV of 81.6% (95% CI 67.5–90.8%). The PLR and NLR were 8.5 (95% CI 3.3–22.0) and 0.2 (95% CI 0.1–0.4). Sensitivity was also higher in sputum (31/37, 83.8%, 95% CI 67.3–93.2%), although specificity was lower (22/25, 88.0%, 67.7–96.8%). No MDR/RR-TB cases were detected from BALF specimens, yielding 94.7% specificity (18/19, 95% CI 71.9–99.9%) (Figure 3a,b, Supplementary Figure S2).

Notably, several discrepant results were observed. Eight MDR/RR-TB cases detected by Xpert were missed by the CRISPR assay (Figure 3a). Three were BALF specimens in which *Mtb* was not detected by the initial qPCR step; another five samples showed phenotypic resistance on pDST, proving false-negative results from the assay. For the five samples, R(20)/R(1) values approximated the threshold, suggesting equivocal signals (Figure 3c). The tandem qPCR–CRISPR assay identified resistance in nine Xpert-negative cases, of which five were concordant with pDST and two were discordant; the remaining two cases were inconclusive because pDST results were unavailable (Figure 3a).

## 4. Discussion

This study presented a preliminary clinical evaluation of a developing tandem qPCR–CRISPR assay for *Mtb* detection and drug resistance identification. The assay exhibited suboptimal performance in both TB diagnosis and drug resistance profiling, with further analyses identifying several areas requiring optimisation.

The tandem qPCR–CRISPR assay detected several smear-negative and culture-negative TB cases, with a high PPV and PLR indicating accuracy in identifying patients with a high probability of disease. However, the moderate NPV limited its utility as a screening tool. Sensitivity did not reach the levels reported for other CRISPR-based assays [14,15,21]. According to the WHO target product profiles (TPPs) for rapid molecular diagnostics, a minimum sensitivity of 80% compared with culture is recommended, with 60% for smear-negative TB and 99% for smear-positive TB. In this study, the assay nearly approached these minimum requirements but failed to meet the target in smear-negative TB cases, suggesting inadequate detection performance in paucibacillary specimens.

This limitation is likely attributable to the serial design of the assay, in which detection relies solely on qPCR without leveraging CRISPR-mediated cleavage activity. In addition, manual nucleic acid extraction may restrict the effective input volume, thereby further compromising sensitivity. Previous studies have reported the sensitivity constraints of qPCR-dependent workflows for TB diagnosis [22,23]. Such workflows are theoretically inferior to Xpert, consistent with our findings. By contrast, CRISPR-based platforms incorporating collateral cleavage activity with amplification have achieved single-copy detection in sealed vessels [24,25]. Furthermore, one-tube formats enabling simultaneous detection of *Mtb* and drug resistance have also been reported [26,27]. Such integrated designs should be prioritized to enhance analytical performance and facilitate clinical deployment. In addition, the qPCR component of the assay targets *IS6110* only, rather than the dual *IS6110*/*IS1081* target strategy employed by Xpert MTB/RIF Ultra. *IS6110* is present in most *Mtb* strains with multicopy, but some strains have been found to have little or no such insertion sequence [28], which can lead to false negative results. The inclusion of *IS1081*, a multi-copy element present in all members of the *Mtb* complex, could enhance detection robustness, including for strains lacking *IS6110*, and reduce the false negative rate. SHINE-TB, for example, couples recombinase polymerase amplification with Cas12a and Cas13a in parallelised one-pot reactions targeting both *IS6110* and *IS1081*, reaching a limit of detection of 69 CFU/mL in spiked sputum while remaining compatible with lateral flow and lyophilisation [25].

Notably, we found that sputum specimens showed higher sensitivity than BALF in the tandem qPCR–CRISPR assay, which contrasts with findings from most previous studies [29,30,31,32]. The reason for the discrepancy may be attributed to suboptimal BALF specimen quality or reduced compatibility of the extraction protocol with non-sputum samples, as supported by the significantly higher Ct values of the internal control observed in BALF compared with sputum specimens.

The tandem qPCR–CRISPR assay showed potential for rapid drug resistance detection, identifying resistance-associated mutations even in several Xpert-negative samples. This finding underscores the contribution of CRISPR-mediated signal amplification for low-abundance targets. For rifampicin resistance, a PLR of 8.5 raises the probability of resistance from the 47.6% observed in our referral cohort to 88.6% after a positive result, but an NLR of 0.2 leaves an 18.4% residual probability after a negative result, far above the threshold at which a clinician could withhold second-line therapy. In a community setting with a 5% pre-test probability the same ratios yield a post-test probability of only 31% after a positive result and 1% after a negative one. Because predictive values depend on prevalence, the PPV reported here would fall substantially in lower-prevalence settings. Against the WHO TPPs thresholds of 95% sensitivity and 98% specificity for rifampicin resistance, the assay met none of the resistance criteria and only marginally approached the detection criterion. We therefore consider the assay, in its current form, an adjunct that may add value in Xpert-negative or Xpert-inaccessible specimens rather than a replacement for Xpert. The assay targets only three key *rpoB* codons, which harbour mutations in approximately 70–90% of resistant strains [17,19,33,34,35], consistent with the detection performance observed in this study. Consequently, rare mutations outside these regions, such as those at codons 511, 513, 522, and 533 [33,34], may lead to missed detection. This contrasts structurally with Xpert MTB/RIF Ultra, whose five overlapping molecular beacon probes span the entire 81-bp region from codon 507 to 533 and call resistance on loss of any probe signal, thereby detecting mutations irrespective of their identity within that window. Interrogating three discrete codons with mutation-specific crRNAs leaves the assay blind to everything between them, which offers the most parsimonious explanation for the sensitivity gap observed against Xpert. The assay also fails to cover mutations that lie outside the 81-bp region and therefore escape Xpert and line probe assays as well, such as I491F and V170F. Both are rising in frequency in parts of Africa, Europe, and Asian countries [36,37]. Incorporating them would represent a genuine advantage over currently deployed assays. Expanding mutation coverage may improve performance, as demonstrated by a next-generation CRISPR assay named FLASH-TB, which targets 52 resistance-associated genes with high sensitivity [38].

Because the share of resistance explained by these three codons varies with lineage and geography, diagnostic performance may differ in other epidemiological settings. Codon 531 accounts for approximately 37% to 70% of resistant isolates across regions [39,40,41,42]. Similarly, codon 526 mutations exhibit frequencies of nearly 40% in China [43], while frequencies are only around 3% in South Africa [44]. Consequently, the diagnostic sensitivity of the assay for rifampicin resistance would be expected to fall further in settings where non-canonical or borderline mutations are more prevalent.

In addition to limited mutation coverage, heteroresistance may represent a plausible biological explanation for false-negative results [45,46]. When resistant bacilli constitute a minority population, mixed infections may generate insufficient fluorescence signal to exceed the predefined threshold. Under such circumstances, the fixed cutoff [R(20)/R(1) ≥ 4.0] defined by the manufacturer may further reduce sensitivity, potentially failing to capture weak but biologically meaningful signals from low-frequency resistant variants. Discrepant cases clustered near the threshold, suggesting that heteroresistance and fixed thresholding together reduce sensitivity when resistant bacilli are scarce. These findings highlight the potential value of adopting adaptive or kinetics-based thresholding strategies in the future optimization of the assay. Low bacterial load, suboptimal specimen quality, limited mutation coverage, and analytical sensitivity constraints (LOD of 100 CFU/mL for resistance mutations) may also contribute to discordance, particularly in paucibacillary specimens where bacterial concentration approaches the detection threshold.

This study has several limitations. First, the generalizability of the findings is constrained by the modest sample size and the absence of extrapulmonary [47] or paediatric TB cases [48,49], populations in whom diagnosis remains particularly challenging. In addition, the single-center design further limits external validity. All participants were enrolled at a single tertiary referral hospital in Shenzhen, China. As demonstrated by the geographical heterogeneity of *rpoB* mutation frequencies across different regions, performance in our cohort may not be representative of other districts. Large-scale, multicenter studies including paediatric and extrapulmonary TB cases will be essential to fully confirm its clinical utility and operational feasibility. Second, targeted *rpoB* sequencing of the discordant specimens could not be undertaken because residual material had been consumed by reference testing. We were therefore unable to determine whether the false-negative results reflected mutations outside the targeted codons or analytical failure. Sequencing of all discordant specimens is a required element of the next validation study. Third, the limited number of BALF specimens and the absence of a sample-specific extraction protocol restrict robust performance analysis, warranting further optimization and validation of the assay across diverse specimen types from larger cohorts.

## 5. Conclusions

The evaluated tandem qPCR–CRISPR assay showed suboptimal performance for the diagnosis of TB and MDR/RR-TB. Further optimization of the assay design, together with integration of more efficient nucleic acid extraction strategies compatible with diverse specimen types, will be essential to meet clinical diagnostic requirements.

## Figures and Tables

**Table 1:** Baseline characteristics and demographic data of participants.

Characteristics ^a,b^	All N = 179	Confirmed TB N = 84	Clinically Diagnosed TB N = 18	Non-TB N = 77	*p*-Values ^c^
Demographics					
Age in years, median (IQR)	52.0 (35.0–60.0)	53.0 (40.3–61.0)	49.0 (34.3–59.3)	51.0 (32.5–58.5)	0.435
Sex					0.003
Male	122 (68.2%)	67 (79.8%)	13 (72.2%)	42 (54.6%)	
Female	57 (31.8%)	17 (20.2%)	5 (27.8%)	35 (45.5%)	
Clinical signs and symptoms					
Cough > 2 weeks	111 (62.0%)	67 (79.8%)	11 (61.1%)	33 (42.9%)	<0.001
Fever	51 (28.5%)	25 (29.8%)	5 (27.8%)	21 (27.3%)	0.938
Expectoration	105 (58.7%)	59 (70.2%)	10 (55.6%)	36 (46.8%)	0.010
Dyspnea	43 (24.0%)	20 (23.8%)	3 (16.7%)	20 (26.0%)	0.765
Fatigue	26 (14.5%)	14 (16.7%)	1 (5.6%)	11 (14.3%)	0.615
Chest pain	12 (6.7%)	11 (13.1%)	1 (5.6%)	0 (0.0%)	0.002
Weight loss	32 (17.9%)	27 (32.1%)	1 (5.6%)	4 (5.2%)	<0.001
Hemoptysis	27 (15.1%)	16 (19.1%)	0 (0.0%)	11 (14.3%)	0.110
Radiographic abnormalities	147 (82.1%)	82 (97.6%)	15 (83.3%)	50 (64.9%)	<0.001
Cavitary lesions	51 (28.5%)	46 (54.8%)	2 (11.1%)	3 (3.9%)	<0.001
Laboratory Investigations ^d^					
Positive IGRA	96 (53.6%)	65 (77.4%)	10 (55.6%)	21 (27.3%)	<0.001
Positive smear	48 (26.8%)	48 (57.1%)	0 (0.0%)	0 (0.0%)	<0.001
Sputum	37 (20.7%)	37 (44.0%)	0 (0.0%)	0 (0.0%)	
BALF	11 (6.1%)	11 (13.1%)	0 (0.0%)	0 (0.0%)	
Positive culture	67 (37.4%)	67 (79.8%)	0 (0.0%)	0 (0.0%)	<0.001
Sputum	52 (29.1%)	52 (61.9%)	0 (0.0%)	0 (0.0%)	
BALF	15 (8.4%)	15 (17.9%)	0 (0.0%)	0 (0.0%)	
Positive Xpert	78 (43.6%)	78 (92.9%)	0 (0.0%)	0 (0.0%)	<0.001
Sputum	59 (33.0%)	59 (70.2%)	0 (0.0%)	0 (0.0%)	
BALF	19 (10.6%)	19 (22.6%)	0 (0.0%)	0 (0.0%)	

^a^ A diagnosis of bacteriologically confirmed TB was assigned to individuals with at least one positive result on smear microscopy, mycobacterial culture, or Xpert testing. Clinically diagnosed cases were active TB patients diagnosed by a clinician based on radiographic abnormalities, suggestive histology, and extrapulmonary evidence without microbiological verification. Non-TB controls were recruited from suspected cases who exhibited no clinical or laboratory evidence of tuberculosis. ^b^ Values are numbers (percent) unless otherwise indicated. ^c^ *p*-values were calculated using the Kruskal–Wallis test for quantitative variables with non-normal distribution, and the chi-square or Fisher’s exact test (two-sided) for categorical variables. ^d^ This denotes the percentage of patients with positive sputum or BALF results relative to the total study population. Definition of abbreviations: TB = Tuberculosis; IQR = Interquartile range; IGRA = Interferon-Gamma Release Assay; BALF = Bronchoalveolar lavage fluid; Xpert = GeneXpert MTB/RIF Ultra.

**Table 2:** Sensitivity of tandem qPCR–CRISPR assay for TB specimens.

Subgroup		qPCR-CRISPR for TB Cases ^a^		Sensitivity (%, 95% CI)	qPCR-CRISPR for Non-TB Cases		Specificity (%, 95% CI)
		Positive	Negative		Positive	Negative	
All Cases		72	30	70.6 (60.6–79.0)	3	74	96.1 (88.3–99.0)
Stratified by specimen type							
Sputum		59	17	77.6 (66.4–86.1)	3	64	95.5 (86.6–98.8)
BALF		13	13	50.0 (30.4–69.6)	0	10	100.0 (65.6–100.0)
Stratified by test ^b^							
Smear	Positive	45	3	93.8 (81.8–98.4)	1	56	98.2 (89.4–99.9)
	Negative	22	23	48.9 (33.9–64.0)			
Culture	Positive	60	7	89.6 (79.1–95.3)	1	44	97.8 (86.8–99.9)
	Negative	6	21	22.2 (9.4–42.7)			
Xpert	Positive	69	9	88.5 (78.7–94.3)	0	58	100.0 (92.3–100.0)
	Negative	3	21	12.5 (3.3–33.5)			

^a^ TB cases were determined based on composite reference standard. ^b^ Specificity was calculated for the non-TB patients who underwent the corresponding test. Definition of abbreviations: TB = Tuberculosis; CIs = confidence intervals; BALF = Bronchoalveolar lavage fluid; Xpert = GeneXpert MTB/RIF ultra.

## Data Availability

The original contributions presented in this study are included in the article. Further inquiries can be directed to the corresponding authors.

## Supplementary Materials

The following supporting information can be downloaded at https://www.mdpi.com/article/10.3390/microorganisms14081750/s1, Figure S1: The workflow of the tandem qPCR-CRISPR assay; Figure S2: The results and performance of the tandem qPCR–CRISPR assay in detecting MDR/RR-TB; Table S1: Primers, probes, and crRNAs used in the qPCR-CRISPR assay.
