# Supplementary material for: Clinical Evaluation of a Tandem qPCR-CRISPR Assay for Multidrug-Resistant or Rifampicin-Resistant Tuberculosis: A Prospective Diagnostic Accuracy Study
Source: Microorganisms. 2026 Aug 9;14(8):1750. doi: 10.3390/microorganisms14081750 (PMC13515032; doi:10.3390/microorganisms14081750)
Supplement: Supplementary file 1 [file microorganisms-14-01750-s001.zip › microorganisms-4405501-supplementary.pdf]

**Supplementary Table S1.** Primers, probes, and crRNAs used in the qPCR-CRISPR assay.

| Primer ID | Sequences (5'->3') |
|---|---|
| FAM-8C-BHQ1 | CCCCCCCC |
| *rpoB*-F | GATCACACCGCAGACGTTGA |
| *rpoB*-R | CGTCGCGGACCTCCAG |
| *IS6110*-F | ACCTATGTGTCGACCTGGGC |
| *IS6110*-R | CAGATGGCTTGCTCGATCGC |
| *IS6110* P-VIC | TTCCACGATGGCCACCT |
| BS-F | CCCTTATGACCTGGGCTACA |
| BS-R | TGTGGGATTGGCTTAACCTC |
| BS-P | AACAAAGGGCAGCGAAACCG |
| sy516T1 | AAUUUCUACUGUUGUAGAUAUGGUCCAGAACAACCGGCA |
| sy526T | AAUUUCUACUGUUGUAGAUCAGUCGGCGCUUGUAGGACA |
| sg526CGC19 | AAUUUCUACUGUUGUAGAUCGCUUGCGGCUCAACCCCG |
| sy526g21 | AAUUUCUACUGUUGUAGAUCAGUCGGCGCUUGUCGGACAA |
| sy531G | AAUUUCUACUGUUGUAGAUGCCCCAGCGCCCACAGUCGG |
| sg526CTC | AAUUUCUACUGUUGUAGAUCAGUCGGCGCUUGAGGGUCU |
| sg531T | AAUUUCUACUGUUGUAGAUCUGUUGGCGCUGGGGUCCGC |

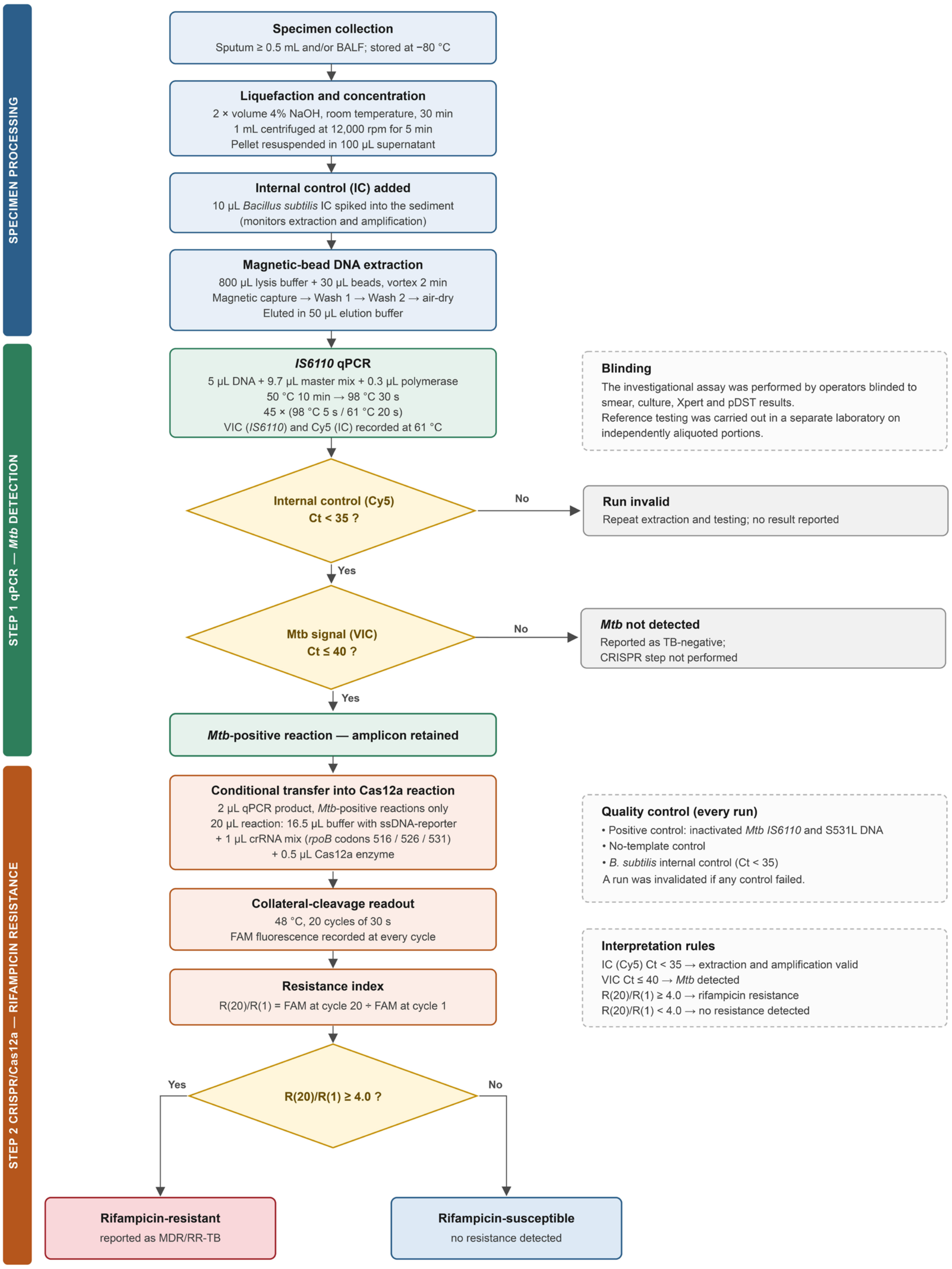


**Supplementary Figure S1.** The workflow of the tandem qPCR-CRISPR assay. Definition of abbreviations: BALF = Bronchoalveolar lavage fluid; Xpert = GeneXpert MTB/RIF Ultra; pDST = Phenotypic drug susceptibility testing; Ct = cycle threshold; TB =Tuberculosis; MDR/RR-TB = Multidrug-resistant or rifampicin-resistant TB.

| Cohort | Count | Rifampicin-resistant with CRISPR | Rifampicin-susceptible with CRISPR | TB-negative with qPCR | Sensitivity (95% CI) | Specificity (95% CI) | PPV (95% CI) | NPV (95% CI) |
|---|---|---|---|---|---|---|---|---|
| Complete | 84 | 35 | 36 | 13 | 77.5 (61.1-88.6) | 90.9 (77.4-97.0) | 88.6 (72.3-96.3) | 81.6 (67.5-90.8) |
| Sputum | 62 | 34 | 24 | 4 | 83.8 (67.3-93.2) | 88.0 (67.7-96.8) | 91.2 (75.2-97.7) | 78.6 (58.5-91.0) |
| BALF | 22 | 1 | 12 | 9 | 0.0 (0.0-69.0) | 94.7 (71.9-99.7) | 0.0 (0.0-94.5) | 85.7 (62.6-96.2) |

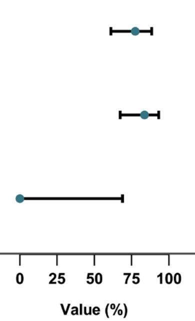


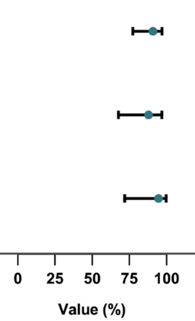


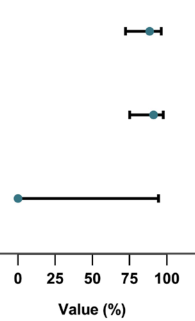


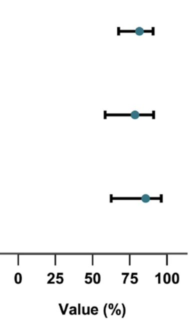


**Supplementary Figure S2.** The results and performance of the tandem qPCR–CRISPR assay in detecting MDR/RR-TB. Definition of abbreviations: BALF = Bronchoalveolar lavage fluid; PPV = positive predictive value; NPV = negative predictive value; CI = confidence intervals.
